# Poetry as therapy

**DOI:** 10.4103/0019-5545.37377

**Published:** 2007

**Authors:** T. M. Raghuram

In this issue we look at poetry. The shared realms of poetry and psychology are vast. Poetry facilitates therapy in myriad ways. Poet psychiatrist Dr. T. M. Raghuram writes an introductory piece on this aspect. A guest poet Roopa uses poetry to sublimate crises (not necessarily her own). Reflective pieces on poetry and the mind are welcome.

**- Ajit V. Bhide**, Literary Editor, (E-mail: mukunjit@yahoo.com)

Poetry is the earliest form of oral literature – a blend of language and music. Historically human beings expressed their emotions through music, from lullabies and love songs to dirges and lamentations. Add to this, ideas and images; and poetry is born. Poetry is also a means of sublimating one's romantic, aggressive or even nihilistic impulses. It acts as a catharsis, providing relief or therapy, so to say. Richard Milne describes the poet as the '. sacred historian of the heart / and moral nature's lord.'. Like in dream analysis, an intuitive psychiatrist can delve into the mind of the poet by sifting through the maze of phrases, metaphors, images and symbols. Some people write in agony and frustration, some in deep love and ecstasy or seething rage. An analysis of the poem gives a picture of the poet's heart.

Poetry communicates at the subconscious level, which is why some lines move us to tears or lift us in joy, inspire us or save us from destroying ourselves. Coleridge used his poetry to soothe the pain of his cancer. Patients with a literary flair can utilize poetic methods to reveal their complex thought processes and conflicts. It helps them to define and grapple with their strange problems. When the psychiatrist has a poetic mind, he can easily make sure of the patient's inner turmoil that is expressed through poetry. When the poet is also a psychiatrist, like William Carlos Williams, we get literary gems out of this amalgamation. To the poetic therapist beset with mind-boggling abnormal predicaments, poetry is both rewarding as well as self therapeutic.

Dr. T. M. Raghuram, M.D. D.P.M.(Vellore), is Asst. Prof. of Psychiatry at M.E.S. Medical College, Perintalmanna, Kerala. He is a well-known Indo-Anglian poet with three published volumes of verse. His first book was shortlisted for the Commonwealth Poetry Prize in 1987. He received the International Poet of Merit Award from USA in 1995. E-mail: tmraghuram@gmail.com - Ajit V. Bhide, Literary Editor.

## A POEM BY ROOPA

I have found my garden
And the sown seeds have grown.
It's the greenness of the leaves
That are beckoning me.
Like the shy moon, I have wanted all day
I have spoken all I could
With thoughts clear as dew drops
Read my hidden expression
Some times behind my frowning furrows.
Don't make me strain harder
Open up and listen
I am chirping louder than the birds
Its my chance to show
Watch me I'm beaming like the moon
Because back home there are people
Waiting to be told
That I have achieved more than I thought I could
I have to carry that trophy back home
Listen, I'm here to stay.

I tried telling you my
Story yesterday
But my emotions choked me.
All I could do was wait for my
Cheeks to dry
Today I can fight back
My tears
Battling within my eye;
I have gathered strength
And courage and can think of words.
Can you understand me,
Watching my quivering lips,
Understand what I'm trying to say ?

It took me all these years
Only to realize that
All my stumbling blocks
Were stepping stones
Today I can relive my past
And pluck away
All those thorns easily
Without my fingers pricked
I now realize
That the path has always been clear
Only I wasn't ready
And I have spent
All my yesterdays
Waiting for today

Tell me If I can help you
Come closer to your dreams
Even if by an inch or two

Some times I think
Time has made me wait
So long
To give me my today
For time
My yesterday is a short while
From its eternity
But I know
I cannot count on my morrows
And today is all I have.

Realize that
A sculptor can give life
And create beauty from stones
We can also sculpt
People around us
Waiting to be sculpted

Don't worry
I will not point
My finger at you
For my arriving late.
I am working
At my short comings
To live my day
Thank you
For bringing me
Closer to myself

In the realms of yesterday
All that happened
Were only noises
Distracting the truth…
I have to some how
Find truth today

